# Interaction Between Heavy Metals Posed Chemical Stress and Essential Oil Production of Medicinal Plants

**DOI:** 10.3390/plants13202938

**Published:** 2024-10-20

**Authors:** Katalin Hubai, Nora Kováts

**Affiliations:** Centre for Natural Sciences, Affiliation University of Pannonia, P.O. Box 158, 8200 Veszprém, Hungary; hubai.katalin@mk.uni-pannon.hu

**Keywords:** medicinal plants, essential oil production, soil pollution, atmospheric pollution, heavy metal stress

## Abstract

Plants exposed to abiotic stressors show diverse physiological, biochemical, and molecular responses. Biosynthesis of plant secondary metabolites—including essential oils—is a vital plant defense mechanism. As these bioactive compounds are widely used in the pharmaceutical, cosmetic and food industries, it is essential to understand how their production is affected in various environments. While interaction between specific abiotic stressors such as salt stress has been widely studied, relatively less information is available on how essential oil production is affected by toxic contaminants. Present review intends to give an insight into the possible interaction between chemical stress and essential oil production, with special regard to soil and air pollution. Available studies clearly demonstrate that heavy metal induced stress does affect quantity and quality of EOs produced, however, pattern seems ambiguous as nature of effect depends on the plant taxon and on the EO. Considering mechanisms, genetic studies clearly prove that exposure to heavy metals influences the expression of genes being responsible for EO synthesis.

## 1. Introduction

Heavy metal (HM) contamination of agricultural soils has become a major environmental concern as potentially toxic compounds reduce the productivity of plants and pose environmental health risks [1]. Heavy metals and metalloids are persistent pollutants because they can accumulate in the soil if they are not taken up by plants or removed by leaching [2]. Most frequently reported HMs include As, Cd, Cr, Cu, Hg, Ni, Pb, and Zn [3]. Several heavy metals are essential for plant life in lower concentrations but become toxic at higher quantities. Examples include B, Cu, Fe, Mg, Mn, Mo, Ni, and Zn [4]. On the other hand, some HMs are highly toxic at almost all concentrations, including As, Cd, Cr, Hg, and Pb [5].

Possible sources of soil pollution include industrial activities, fossil fuel burning, traffic, mining, smelting, waste or wastewater disposal [6]. In addition, heavy metal pollution can occur during agricultural practice, such as using heavy metal-enriched organic additives or via irrigation using wastewater containing heavy metals in high concentrations [7].

A wide range of studies have reported that medicinal plants are exposed to heavy metal pollution in their natural habitats, making collection and use for human consumption unsafe. Karahan et al. reported increased heavy metal concentrations in medicinal plants collected from Eastern Mediterranean Region of Turkey in habitats being impacted by industrial activity or mining [8]. Atmospheric heavy metal contamination was monitored in the study of Agoramoorthy et al. [9] in halophytic medicinal plants in Tamil Nadu (India), demonstrating actual impact. Tomaszewska-Sowa et al. assessed heavy metal load and subsequent accumulation of heavy metals in natural habitats in Poland [10]. The study reported that some metals, e.g., Pb and Hg were found in elevated concentrations in rhizopheric soils and in some medicinal plants. Lead contamination was also found in natural habitats in Serbia [11].

Considering the fact that medicinal plants are of global value [12], a wide range of studies have been targeted to examine the relationship between heavy metal contamination and plants’ responses. Most of the studies concentrate on possible accumulation of HMs in the plants’ body [13], as it has been widely assumed this in turn poses human health risks on consumers [14].

On the other hand, the response of plants is also of crucial importance. Secondary metabolites (SMs), including essential oils, are playing a vital role in the plant’s defense against environmental stressors, like chemical agents [15,16]. It is generally hypothesized that heavy metal stress triggers the production of SMs [17]. Recent review has been written with the aim to summarize studies and findings which enlighten the potential impact of heavy metals posed chemical stress on the production and metabolism of essential oils.

## 2. Exposure Routes

HMs can enter the plants’ body via both root and foliar uptake. Comparing the two exposure pathways, the soil-root transfer is the major process determining metal concentration in the different organs of the plant [18]. In areas affected by industrial activity, mining or high traffic, uptake via both pathways can occur simultaneously. However, the soil-root transfer has been more thoroughly studied than the air-leaves-stem pathway [19,20].

### 2.1. Uptake from the Soil

HMs being present in the soil are first adsorbed onto the root surfaces, followed by the passive penetration and diffusion along with water molecules. Another possible process implies active transfer along a concentration gradient [21].

Soil physico-chemical properties such as soil particle size, cation exchange capacity affect metal availability to a great extent. Soil pH influences the solubility, bioavailability and therefore, toxicity of HMs. At low pH, metals rather occur in more bioavailable free ionic forms, at higher pH they form phosphates and carbonates which are barely soluble [22]. Microbial activity can also modify the biosorption and bioavailability of heavy metals [23].

Partitioning between organs will also influence the presence and potential toxic effects of HMs. Olowoyo et al. measured the concentrations of different heavy metals (Fe, Mn, Zn, Cu, Cr, Ni and Pb) in the roots, stems and leaves of *Datura stramonium* and *Amaranthus spinosus*, reporting a diminishing order of all elements in the root-stem-leaves route [24]. Similar pattern was reported by Tripathi et al. [25]. Heavy metals, however, can be translocated to above-ground parts. Pehoiu et al. [26] measured heavy metal uptake and translocation in medicinal plants such as *Plantago major*, or *Taraxacum officinale*, and found high amounts of Cd, Mn and Pb in leaves.

### 2.2. Foliar Uptake

A relatively scarcely discussed exposure pathway is airborne contamination. Atmospheric particulate matter (PM) is grouped according to aerodynamic diameter of particles as coarse, fine, and ultrafine particles (UFPs) with aerodynamic diameters of 2.5 to 10 μm (PM10), <2.5 μm (PM2.5), and <0.1 μm (PM0.1), respectively. While PM10 and PM2.5 fractions are better known and characterized, UFP fate, composition and toxicity has become an emerging issue quite recently. Particle size is an important aspect, as PM might bind potentially toxic chemicals, including heavy metals. The smaller the more hazardous: as fine particles have relatively bigger surface, they can bind relatively higher amount of contaminants. In a size-fractionated urban sample, Srivastava and Jain [27] demonstrated that most of the metal mass (Mn, Cr, Cd, Pb, Ni, and Fe) were concentrated in the PM0.7 fraction. Hu et al. assessed the foliar uptake of lead from nine-stage size-segregated aerosols and found that the fine fractions enriched more Pb than the coarse fractions [28].

Potential sources of atmospheric heavy metals include natural (such as forest fires) as well as anthropogenic sources (mining, waste incineration, etc.) [29]. Another important source of is brake dust [30]. Of potentially toxic elements, Pb and Cd are still dominant components of atmospheric aerosol [31]. The presence of redoxiactive trace elements such as Ni, Cr, Co, and As also contribute to the potential toxicity of atmospheric PM [32]. However, heavy metal content of atmospheric PM shows a seasonal pattern; for example, Pb, which is primarily derived from the anthropogenic source, occurs in elevated concentrations in spring and winter [33].

In comparison to the soil-to-root transfer, uptake of heavy metals from atmospheric fallout has been much less studied [34]. Plants are exposed to airborne particulates via dry or wet deposition; in the latter case, particles are washed out by rain, snow or fog. Pan and Wang [35] compared the significance of dry and wet deposition fluxes for a wide range of heavy metals and found that for most of them, dry deposition was the dominant process. However, in case of some HMs such as nickel, arsenic, lead, zinc, cadmium, the relative contribution of wet and dry deposition fluxes varied by sampling sites. Wet deposition, in general, strongly depends on the local pattern of precipitation [36] and shows seasonal variability [37].

Metals contained in the deposited particles can be taken up via internalization through the cuticle and penetration through stomatal openings [38]. Different species will show varying efficiency in absorbing HMs as leaf morphology significantly influences the extent plants capture and retain PM [39]. Most important morphological features are leaf size and shape [40], roughness, the presence or absence of leaf hairs [41] and the presence and thickness of epicuticular way layer [42].

## 3. Potential Toxic Effects of HMs

According to Feki et al. [43], phytotoxic effects of HMs are grouped as (1) physiological responses, including the inhibition of photosynthesis, as well as plant growth inhibition; (2) biochemical responses, including producing reactive oxygen species, resulting in oxidative stress and modification of the antioxidant enzymes activities and (3) molecular effects, represented basically by the modification of the expression of metals stress responsive genes.

The most generally reported symptom in heavy metal stressed medicinal plants is reduced growth (reviewed by Maleki et al. [44]). Growth impairment can be associated with the damage of photosynthetic processes. Also, the presence of heavy metals can reduce nutrient availability: Al-Rashedy for example experimentally demonstrated in soils treated with cobalt and nickel, uptake of sodium and potassium decreased in spearmint (*Mentha spicata* L.) [45]. Nutrient deficiency, naturally, can result in lower growth rate.

Sytar et al. [46] discuss that the inhibition of photosynthesis is one of the major effects, affecting both light and dark reactions. Toxicity symptoms of HM exposure involve the decline of photosynthetic activity and decrease in the photosynthetic pigments’ (chlorophyll a, b and carotene) concentrations [47]. The potential harmful effects of HM pollution were depending on the length of exposure in the experiment of Dinu et al. [48]. Mint seedlings were exposed for two metal mixtures (As+Cd and As+Cd+Ni+Pb). After one month of exposure, test plants showed an increased chlorophyll content in the As+Cd+Ni+Pb treatment, but the tendency changed by the end of the total exposure of three months. Symptoms of phytotoxicity, such as chlorosis and leaves’ loss were detected.

One major mechanism of HMs phytotoxicity is the overproduction of reactive oxygen species (ROS) which induce oxidative stress in plants. Plants have developed an efficient antioxidative defense system that includes both enzymatic components such as superoxide dismutase, catalase, peroxidases, and glutathione reductase as well as non-enzymatic ones such as ascorbic acid (reviewed by Mansoor et al. [49]).

The levels of these enzymes are expected to increase in case of HM induced stress. Biswas et al. [50] experimentally showed the increased levels of antioxidant enzyme activities such as guaiacol peroxidase (GPX), superoxide dismutase (SOD) and ascorbate peroxidase (APX) in the medicinal plant *Centella asiatica* (L.) Urban (asiatic pennywort or brahmi) cultivated in soils polluted with cadmium and lead. However, in case of excess contamination load, the activity of some enzymes involved in defense responses can be disrupted [51]. Increase in the activity of non-enzymatic antioxidants under heavy metal stress was also reported, as part of detoxification processes [52,53].

## 4. Effects of Heavy Metals on Essential Oil Production

The number of studies specifically targeted to assess EO concentrations and/or composition in plants exposed to heavy metal stress is rather limited. The following chapter intends to give a summary of these works. In laboratory experiments, performance of test plants was assessed in different cultivating media, with the emphasis of experimentally treating soil with a pre-set concentration series of HMs or their combination. Lab-scale studies also include the assessment of potentially toxic soil samples brought to the laboratory. Actual field studies where toxic symptoms of essential oil-bearing plants grown in contaminated environments are evaluated will be discussed separately.

### 4.1. Lab-Scale Studies

Most studies address cadmium (Cd), lead (Pb), and copper (Cu) as they are considered the most widespread heavy metal contaminants in agricultural soils [54]. Pirooz et al. [55] (2022) treated 50-day-old sage (*Salvia officinalis* L.) plantlets with various concentrations of CuSO_4_ (0, 100, 200, 400, and 800 μM). CuSO_4_ was added to half-strength Hoagland’s solution used for watering the plantlets. Treatment up to 200 μM elucidated the increase in total essential oil production. Similarly, watering with 40 mM solution of CuSO_4_ was used in the study of Es-sbihi et al. [56]. This concentration was preliminary assessed and elucidated phytotoxicity, resulting in growth inhibition. Treated *S. officinalis* plants showed significant (16.66%) increase in EO yield as compared to control.

Elzaawely et al. [57] experienced reduction in the total EO yield when shell ginger (*Alpinia zerumbet* (Pers.) B.L. Burtt. & R.M. Sm., family Zingiberaceae) plants were sprayed with 500 mM copper sulphate (CuSO_4_), though some components showed increase such as 1,8-cineol, linalool, camphor, borneol, or cuminaldehyde. Moderate Cu and Zn treatment had a stimulating effect on major components of EO in pennyroyal (*M. pulegium* L.), namely pulegone, cis-isopulegone, a-pinene, sabinene, 1,8-cineol and thymol [58].

Babashpour-Asl et al. [59] used irrigation with Cd solution (4 mg/L, and 8 mg/L, 1250 mL in each pot) to treat coriander (*Coriandrum sativum* L.) and found that the lower Cd concentration (4 mg/L) increased EO production.

Different treatment method was used in the study of Fattahi et al. [60], where soil was pre-treated using Cd (0, 5, 10, 20 mg/kg soil) and Pb (0, 100, 200, 400 mg/kg soil). Both Cd and Pb had a stimulating effect on total EO production of the test plant, sweet basil (*Ocimum basilicum* L.). However, when analyzing the effect on the composition of EOs, the authors found that both Cd and Pb increased the concentration of some EOs such as octanol, linalool while decreased other compounds like α-pinene. Similarly, stimulating effect was demonstrated in the study of Poursaeid et al. [61]. EO yield, especially main compounds of the essential oil like estragole, linalool and geranial of *O. basilicum* increased upon the treatment with 25, 75, 100, and 150 µM Cd in a dose-dependent manner.

Mohammed et al. [62] irrigated different mint cultivars (peppermint, *Mentha x piperita* L. and curly mint, *M. spicata* var. *crispa* L.) with different concentrations of Cd reaching soil levels of 15, 30, and 45 mg/kg. In case of 15 mg/kg level, EO production increased in both test plants. All treatments, however, triggered phytotoxic effects such as growth impairment and reduction in total chlorophyll content. Cd and Pb also elucidated higher EO production in *O. basilicum* L. in the study of Youssef [63], upon treatment with 5, 10, 15, 20, 25 ppm Cd and 100, 350, 750, 1000, 1500 ppm Pb. On the contrary, lemon balm test plants (*Melissa officinalis* L.) were exposed to 10, 20, and 30 mg/kg of soil Cd in a chronic, 3 months test [64]. In addition to growth and morphological impairments, EO production significantly decreased. Authors concluded that both structural and functional damage could have damaged EO producing mechanisms.

Irrigation with Cd and Pb was used for treatment in the study of Amirmoradi et al., in concentrations 10, 20, 40, 60, 80, 100 ppm for Cd and 100, 300, 600, 900, 1200, 1500 ppm for Pb [65]. Essential oil content of the test plant *M. piperita* decreased with increasing concentrations of Cd and Pb, phytotoxic symptoms also appeared. Similar treatments with Cd were applied in the study of Azimychetabi et al., also using *M. piperita* as model plant [66]. Constituents of peppermint oil showed different behavior: pulegone and menthofuran quantities increased, whereas menthol content decreased.

Kunwar et al. [67] applied Pb in 500, 600, 750, 900 mg/kg, Cu in 270, 300, 500, 700 mg/kg and Cd in 6, 10, 20, 30 mg/kg concentrations, also comparing the response of two plants, *M. spicata* and *O. basilicum*. In *O. basilicum*, total EO production increased, including the main component, linalool, while methyl chavicol yield decreased. In case of the other plant examined, *M. spicata,* no significant change was found. Sulastri and Tampubolon [68] also investigated the effect of Cd, using different species (*Vetiveria zizanioides*, *Cymbopogon citratus*, *C. nardus*, *Curcuma xanthorrhiza*, *Pogostemon cablin*, and *Alpinia galanga*). Striking species-dependent differences were found: while *V. zizanioides* showed an app. 100% increase in EO yield, the other species showed much lower or no response.

Sá et al. [69] cultivated spearmint (*M. crispa*) on experimentally treated soil, using 900, 1800, 3600, 7200, and 9000 mg/kg of Pb. At higher Pb levels, essential oil yield significantly increased, also, its chemical composition was affected. The concentration of the major component of *M. crispa* essential oil, carvone, reached as much as 90% in Pb-contaminated soils.

Species-dependent sensitivity was found in the study of Prasad et al. [70]. 30.0 and 60.0 mg/kg soil of chromium and lead concentrations were assessed on the production and chemical profile of essential oil of three *Mentha* species (*M. piperita*, *M. arvensis*, and *M. citrata*). The essential oil yield of *M. arvensis* and *M. citrata* was significantly reduced by both applications while that of *M. piperita* was significantly increased. Zheljazkov et al. [1] prepared soil samples with Cd, Pb, Cu and their combination for cultivation of dill (*Anethum graveolens* L., cv. Hercules), peppermint (*Mentha x piperita* L., cv. Mitchum), and basil (*Ocimum basilicum* L., cv. Broad Leaf Italian). Treatment reduced the menthol content in the peppermint oil and reduced the total oil content in basil, also, copper applied at the highest concentration of 150 mg/L reduced oil content in dill.

Nabi et al. [71] investigated the effects of nickel (Ni) on menthol mint (*M. arvensis* L.). Plantlets were transplanted into soil treated with Ni, the concentrations were set at 20, 40, 60, 80, and 100 mg/kg Ni of soil. A clear Janus-faced effect was experienced: stimulation of essential oil production in case of the lowest (20 mg/kg of soil) concentration but inhibition at higher concentrations. Effect on different EO production, however, was not unambiguous: while production of menthol, the main constituent of mint oil, was already reduced in the lowest concentration as compared to the control, menthone and menthyl acetate concentrations were increased in the lower concentrations.

Arsenic (As) treatment was performed by Biswas et al. [72], applying disodium hydrogen arsenate [Na_2_HAsO_4_·7H_2_O] reaching 10, 50, 150 mg/kg As soil concentrations. Dose-dependent change in total EO production in *O. basilicum* was experienced: increase in 10 and 50 mg/kg concentrations but decrease at the higher, 150 mg/kg concentration. Different EO components showed contradictory tendencies: linalool, the main EO compound increased but others such as 1,8-cineol and methyl eugenol decreased.

Other studies measured EO content in test plants cultivated in soils treated with complex mixture of heavy metals. No change in the essential oil composition was reported by Scora and Chang [73] when peppermint (*M. piperita*) was grown on sewage sludge-treated soils containing Cd, Cr, Cu, Ni, Pb, and Zn. Pandey et al. [74] grew palmarosa (*Cymbopogon martinii* (Roxb.) Wats.) plants (family Poaceae) on tannery sludge polluted soil, potentially exposed to Cr, Ni, Pb and Cd stress. No change was observed in EO yield.

Gautam and Agrawal [75] used lemongrass (*Cymbopogon citratus* (D.C.) Stapf.) (family Poaceae) plants to assess the potential effect of heavy-metal containing red mud. Red mud was mixed with sewage sludge amended soil at different concentrations (5, 10 and 15% *w*/*w*). The two lower doses had positive effect on total EO yield.

### 4.2. Field Experiments

Field experiments have also been conducted, mainly to evaluate if essential oil-bearing plants can be cultivated in areas affected by anthropogenic pollution. These studies compare the general performance of these plants to reference sampling areas, parameters assessed include growth, yield and EO yield. However, number of such studies is very limited, and they cover only a few medicinal plant species. Zheljazkov et al. [76] did not find significant differences between EO content of *Lavandula angustifolia* Mill in field experiments, comparing lavander grown in control site and near a non-ferrous metals combine factory, on heavily polluted soils.

Gharib et al. [77] compared EO content of wild mint (*M. longifolia*) collected from polluted and unpolluted sections of River Nile (Egypt). Not only EO yield was higher in the polluted canals but also, antioxidant activity of EO was more pronounced as measured by the free radical 2,2-Diphenyl-1-picrylhydrazyl (DPPH) assay. However, different components of the EO showed different tendency: plants grown in polluted environment showed increased menthone but decreased pulegone content.

Givianrad and Hashemi [78] measured different components (monoterpene hydrocarbons, oxygenated monoterpenes; sesquiterpene hydrocarbons and oxygenated sesquiterpenes) in *Tanacetum polycephalum* Sch.Bip. (family Asteraceae) grown at increasing differences from Veshnaveh-Qom mine (Iran). With increasing distance, the concentration of each group also increased. Main heavy metals components measured in the soil samples were Cu and Ag.

Table 1 summarizes literature conducted both lab-scale and in the field, assessing the impact of different heavy metals and/or environmental pollution on EO production of different medicinal plants.

### 4.3. Assessment of Atmospheric Heavy Metal Pollution

Basile et al. [79] compared the chemical composition of feijoa (*Feijoa sellowiana* Berg., family Myrtaceae) oils from plants growing in a control site to plants collected from a site exposed to air pollution (situated in Naples, Italy). Antioxidant compounds like limonene, (E)-β-ocimene, α-terpineol, β-caryophyllene, etc. were found in significantly higher concentrations in polluted samples. Judzentiene et al. [80] used the composition of the essential oils in the needles of Scots pine (*Pinus sylvestris* L.) as an indicator to assess the effect of different factories in Lithuania. In general, industrial emissions caused definitive response, but in different ways. Pollution from the oil refinery and the cement factory increased the production of shorter-chain terpenes while the nitrogen fertilizer factory increased the production of longer-chain terpenes.

Nivinskiene et al. [81] analyzed the composition of essential oils of small-leaved linden (*Tilia cordata* Mill.) blossom collected in reference sites vs. in urban environment of Vilnius (Lithuania). The percentage of oxygenated compounds (such as monoterpenes, sesquiterpenes, etc.) was higher in the ecologically clean localities. Hubai et al. [82] treated *O. basilicum* plants with the aqueous extract of atmospheric PM, simulating wet deposition. Eugenol content of the treated sample was not affected, but linalool content showed a statistically significant increase after the treatment.

It should be noted, however, that in urban environments atmospheric particulate matter binds a wide range of potentially toxic compounds in addition to heavy metals. Polycyclic aromatic compounds are the most widely studied, as these compounds have well-documented phyotoxicity and accumulation potential [83]. Plants exposed to atmospheric pollution will most likely respond to the complex mixture of contaminants, in addition to heavy metals.

## 5. Possible Mechanisms

The presence of heavy metals in the medicinal plants’ environment definitely influences essential oil production. However, there is very scarce literature discussing the potential mechanisms behind. Figueiredo et al. [84] suggest that the emission of volatiles is part of the defense mechanisms of plants. In general, secondary metabolites play a well-documented role in counteracting ROS stress [85]. Bibbiani et al. [86] detected a wide range of volatile organic compounds in the volatilome of the mint *Tetradenia riparia* exposed to Zn stress. These VOCs included important essential oils like methanol, considered as adaptive response by the authors.

**Figure 1:**
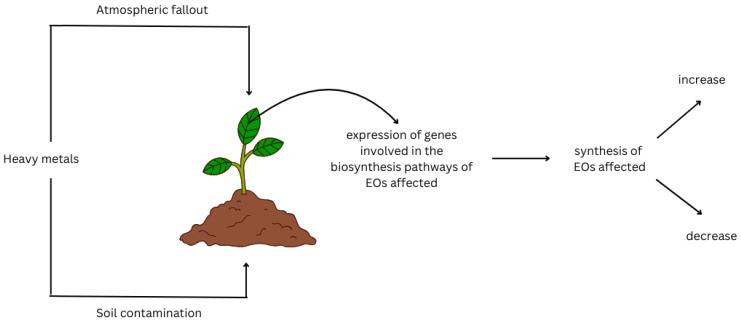
Schematic diagram summarizing the mode of action of heavy metals on EO synthesis.

The most important definite proof is the study of Azimychetabi et al. [66]. *M. piperita* test plants exposed to increasing soil Cd concentrations showed alterations in essential oil content, in parallel with the reduction in the expression of menthone reductase and pulegone reductase genes and increase in the expression of menthofuran synthase. Mode of action of HMs on EO synthesis is illustrated in Figure 1.

## 6. Bioaccumulation

A wide range of studies have investigated the accumulation of different heavy metals in medicinal herbs. Bioaccumulation—the uptake and steady-state storage of contaminants—is considered a serious hazard for potential consumers, including humans. Some studies have even reported human health problems associated with the consumption of HM-contaminated herbal medicines [87]. Chen et al. [88] reviews a wide range of health effects reported in association with the occurrence of selected heavy metals in medicinal herbs. The study summarizes 1902 samples belonging to 118 different medicinal herbs. Several human health issues have been reported according to the review, including (but not restricted to) dermatitis, gastrointestinal symptoms such as bleeding, kidney or liver damage, toxic effects on the immune system. Luo et al. [89] conducted a thorough study analyzing 1773 samples collected worldwide, these samples belonged to 86 different kinds of commonly used herbs. Several toxic metals occurred in over-limit concentrations, such as Pb in 5.75% of the samples, followed by Cd (4.96%), As (4.17%), Hg (3.78%), and Cu (1.75%). Bioaccumulation of hazardous HMs can occur in both herbal plants collected from their natural habitats [90] or cultivated, depending on vicinity to potential pollution sources [91].

Plants have developed tolerance mechanisms to heavy metals taken up from the soil such as exclusion and accumulation (reviewed by Sarma et al. [92]). In the case of accumulated HMs, cells maintain the intracellular heavy metal ions, but in a detoxified form, for example in metal-binding peptides. Exclusion implies that HMs are later removed by leaf fall, therefore plants can get rid of these potentially toxic materials. In addition, so-called excluder plants retain certain HMs such as Pb in the root system preventing further transfer to the shoots [93].

Accumulation of heavy metals in *O. basilicum* has been reported by several authors: Cd, Cr, and Pb [94]; Cd, Cr, Pb, and As [19]. No increased concentration of these metals was documented, however, in the essential oils of such plants [61,95].

Lal et al. [96] assessed essential oil yield, accumulation of heavy metals in lemon grass (*Cymbopogon flexuosus*) cultivated under various irrigation regimes of primary treated wastewater containing Cd, Cr, Ni and Pb. These metals showed a tendency to accumulate in the plants, however, no accumulation was found in the essential oils. Gautam and Agrawal [75] measured heavy metal content of *Cymbopogon citratus* plants and their EOs when test plants were grown on a soil mixture containg red mud in different ratios. While the plants accumulated Fe, Zn, Cu, Cd, Ni and Pb in higher amounts than the safe limit for medicinal plants, no such accumulation was measured in the EO samples.

Lydakis-Simantiris et al. [97] cultivated three medicinal plant species (*Matricaria recutita*, /family Asteraceae/, *Thymus vulgaris* and *Salvia officinalis*, /family Lamiaceae/) in heavy metal polluted soils. The study applied nitrate salts of Cd, Pb, and Ni through irrigation, reaching the final concentrations of the metals in the soil as Cd 1, 3, 10, 30 ppm, Pb 60, 180, 600, 1800 ppm, and Ni 20, 60, 200, 600 ppm. While significant accumulation was experienced in the plant tissues, essential oils remained unaffected.

Zheljakov et al. [1] measured Cd, Pb, Cu levels in EOs of *A. graveolens, M.* x *piperita* and *O. basilicum* and reported no detectable accumulation. Authors also stress that such plants can be grown in Cd, Pb, and Cu enriched soils without significant impairment of essential oil composition and quality. Angelova et al. [98] conducted a field study on an agricultural fields contaminated by metal works near Plovdiv (Bulgaria). Lavander (*Lavandula angustifolia* L.) accumulated Pb, Zn and Cd but these HMs were not transferred in the essential oils. The study proved that lavender can be grown for phytoremediation of contaminated soils.

These studies clearly indicate that although heavy metal accumulation can be a serious risk affecting the consumption of the medicinal herb itself, EOs remain intact. Collection of essential oil-bearing plants often occurs in contaminated habitats, however, their EO yield can be utilized for human consumption. Lydakis-Simantiris et al. [97] suggest that such plants can be successfully grown on heavy metal polluted areas but exclusively for EO production.

## 7. Concluding Remarks

Plants are known to show a wide range of symptoms when exposed to heavy metal stress, from growth impairment to biochemical markers such as changes in antioxidant enzymes concentrations. The production of essential oils, which plays an important part in the plants’ defense mechanisms, seems to be impacted by heavy metals of known phytotoxicity. However, reported studies seem to be ambiguous and no clear tendency can be observed. Changes in the essential oil content of exposed medicinal plants—both in quantitative and qualitative—are strongly dependent on the type of the plant as well as the type of the EO itself. The picture is even more complicated as in some cases, lower HM concentrations increased but high concentrations decreased EO production, suggesting the damage to EO metabolism. Considering the relatively limited number of published works which in turn have targeted a limited variety of medicinal plants, two main conclusions can be drawn now. When mechanisms are to be explained, it has been proven that heavy metals already express their effects on the level of genes being responsible for EO synthesis, influencing the expression of genes. Secondly, several studies addressed in this review have been conducted to assess if medicinal plants used for essential oil production could be cultivated in heavy metal enriched soils providing suitable alternatives to edible crops. Evaluation of bioaccumulation indicates that such plants can take up heavy metals in considerable quantities, but it does not contaminate their essential oils. The main risk, however, is that cultivation of collection of essential oil-bearing plants in contaminated environments will result in the change of composition and/or quantity of their EO yield in an unpredictable way. Unfortunately, the range of plants investigated is small, which makes extrapolations even more limited, also suggesting the gaps further research should fill in.

## Figures and Tables

**Table 1:** Summary of lab-scale and field studies.

Reference	Plant	HM Examined/Nature of Study
Pirooz et al. 2022 [55]	*Salvia officinalis* L.	Cu, lab-scale
Es-sbihi et al. 2020 [56]	*S. officinalis* L.	Cu, lab-scale
Elzaawely et al. 2007 [57]	*Alpinia zerumbet* (Pers.) B.L.Burtt and R.M.Sm.	Cum lab-scale
Lajayer et al. 2017 [58]	*Mentha pulegium* L.	Cu, Zn, lab-scale
Babashpour-Asl et al. 2022 [59]	*Coriandrum sativum* L.	Cd, lab-scale
Fattahi et al. 2019 [60]	*Ocimum basilicum* L.	Cd, Pb, lab-scale
Poursaeid et al. 2021 [61]	*O. basilicum* L.	Cd, lab-scale
Mohammed et al. 2024 [62]	*Mentha piperita* L., *M. spicata* var. *crispa* L.	Cd, lab-scale
Youssef 2021 [63]	*O. basilicum* L.	Cd, Pb, lab-scale
Kilic and Kilic 2017 [64]	*Melissa officinalis* L.	Cd, lab-scale
Amirmoradi et al. 2012 [65]	*M. piperita* L.	Cd, Pb, lab-scale
Azimychetabi et al. 2021 [66]	*M. piperita* L.	Cd, lab-scale
Kunwar et al. 2015 [67]	*M. spicata* L., *O. basilicum* L.	Cd, Cu, Pb, lab-scale
Sulastri and Tampubolon 2019 [68]	*Vetiveria zizanioides* (L.) Nash, *Cymbopogon citratus* (DC.) Stapf, *C. nardus* (L.) Rendle, *Curcuma xanthorrhiza* Roxb., *Pogostemon cablin* (Blanco) Benth., *Alpinia galanga* (L.) Willd.	Cd, lab-scale
Sá et al. 2015 [69]	*M. crispa* L.	Pb, lab-scale
Prasad et al. 2010 [70]	*M. piperita* L., *M. arvensis* L., *M. citrata* Ehrh.	Cd, Pb, lab-scale
Zheljazkov et al. 2006 [1]	*Anethum graveolens* L., *Mentha piperita* L., *O. basilicum* L.	Cd, Pb, Cu
Nabi et al. 2020 [71]	*M. arvensis* L.	Ni, lab-scale
Biswas et al. 2015 [72]	*O. basilicum* L.	As, lab-scale
Scora and Chang 1997 [73]	*M. piperita* L.	Cd, Cr, Cu, Ni, Pb, Zn sewage sludge-treated soils
Pandey et al. 2015 [74]	*Cymbopogon martinii* (Roxb.) Wats.	Cr, Ni, Pb, Cd, tannery sludge polluted soil
Gautam and Agrawal 2017 [75]	*Cymbopogon citratus* (DC.) Stapf.	Red mud mixed with sewage sludge amended soil
Zheljazkov et al. 1996 [76]	*Lavandula angustifolia* Mill.	Field experiment
Gharib et al. 2021 [77]	*Mentha longifolia* (L.) Huds.	Field experiment
Givianrad and Hashemi 2014 [78]	*Tanacetum polycephalum* Sch.Bip.	Field experiment

## Data Availability

No new data were created or analyzed in this study. Data sharing is not applicable to this article.

## References

[1] Zheljazkov V.D., Craker L.E., Xing B. (2006). Effects of Cd, Pb, and Cu on growth and essential oil contents in dill, peppermint, and basil. Environ. Exp. Bot..

[2] Ali H., Khan E., Ilahi I. (2019). Environmental Chemistry and Ecotoxicology of Hazardous Heavy Metals: Environmental Persistence, Toxicity, and Bioaccumulation. J. Chem..

[3] Tóth G., Hermann T., Da Silva M.R., Montanarella C. (2016). Heavy metals in agricultural soils of the European Union with implications for food safety. Environ. Int..

[4] Shahid M., Khalid S., Abbas G., Shahid N., Nadeem M., Sabir M., Aslam M., Hakeem H.R. (2015). Heavy Metal Stress and Crop Productivity. Crop Production and Global Environmental Issues.

[5] Rai P.K., Leeb S.S., Zhang Tsang U.F., Kime K.H. (2019). Heavy metals in food crops: Health risks, fate, mechanisms, and management. Environ. Int..

[6] Zwolak A., Sarzyńska M., Szpyrka E., Stawarczyk K. (2019). Sources of soil pollution by heavy metals and their accumulation in vegetables: A review. Water Air Soil Pollut..

[7] Meng W., Wang Z., Hu B., Wang Z., Li H., Goodman R.C. (2016). Heavy metals in soil and plants after long-term sewage irrigation at Tianjin China: A case study assessment. Agric. Water Manag..

[8] Karahan F., Ozyigit I.I., Saracoglu I.A., Yalcin I.E., Ozyigit A.H., Ilcim A. (2020). Heavy metal levels and mineral nutrient status in different parts of various medicinal plants collected from eastern Mediterranean region of Turkey. Biol. Trace Elem. Res..

[9] Agoramoorthy G., Chen F.A., Hsu M.J. (2008). Threat of heavy metal pollution in halophytic and mangrove plants of Tamil Nadu, India. Environ. Pollut..

[10] Tomaszewska-Sowa M., Kobierski M., Sawilska A.K., Figas A. (2018). Assessment of phytoaccumulation of trace elements in medicinal plants from natural habitats. Herba Pol..

[11] Stankovic D., Krstic B., Orlovic S., Trivan G., Poljak P.L., Nikolic M.S. (2011). Woody plants and herbs as bioindicators of the current condition of the natural environment in Serbia. J. Med. Plants Res..

[12] Chen S.L., Yu H., Luo H.M., Wu Q., Li C.F., Steinmetz A. (2016). Conservation and sustainable use of medicinal plants: Problems, progress, and prospects. Chin. Med..

[13] Rao M.M., KumarMeena A., Galib (2011). Detection of toxic heavy metals and pesticide residue in herbal plants which are commonly used in the herbal formulations. Environ. Monit. Assess..

[14] Vinogradova N., Glukhov A., Chaplygin V., Kumar P., Mandzhieva S., Minkina T., Rajput V.D. (2023). The content of heavy metals in medicinal plants in various environmental conditions: A review. Horticulturae.

[15] Jan R., Asaf S., Numan M., Lubna, Kim K.M. (2021). Plant secondary metabolite biosynthesis and transcriptional regulation in response to biotic and abiotic stress conditions. Agronomy.

[16] Yeshi K., Crayn D., Ritmejerytė E., Wangchuk P. (2022). Plant secondary metabolites produced in response to abiotic stresses has potential application in pharmaceutical product development. Molecules.

[17] Lajayer B.A., Ghorbanpour M., Nikabadi S. (2017). Heavy metals in contaminated environment: Destiny of secondary metabolite biosynthesis, oxidative status and phytoextraction in medicinal plants. Ecotox. Environ. Saf..

[18] Shahid M., Dumat C., Khalid S., Schreck E., Xiong T., Niazi N.K. (2017). Foliar heavy metal uptake, toxicity and detoxification in plants: A comparison of foliar and root metal uptake. J. Hazard. Mater..

[19] Boechat C.L., Carlos F.S., Gianello C., de Oliveira Camargo F.A. (2016). Heavy metals and nutrients uptake by medicinal plants cultivated on multi-metal contaminated soil samples from an abandoned gold ore processing site. Water Air Soil Pollut..

[20] Hlihor R.M., Roșca M., Hagiu-Zaleschi L., Simion I.M., Daraban G.M., Stoleru V. (2022). Medicinal plant growth in heavy metals contaminated soils: Responses to metal stress and induced risks to human health. Toxics.

[21] Maleki M., Ghorbanpour M., Kariman K. (2017). Physiological and antioxidative responses of medicinal plants exposed to heavy metals stress. Plant Gene.

[22] Adamczyk-Szabela D., Wolf W.M. (2022). The impact of soil pH on heavy metals uptake and photosynthesis efficiency in *Melissa officinalis*, *Taraxacum officinalis*, *Ocimum basilicum*. Molecules.

[23] Abdu N., Abdullahi A.A., Abdulkadir A. (2017). Heavy metals and soil microbes. Environ. Chem. Lett..

[24] Olowoyo J.O., Okedeyi O.O., Mkolo N.M., Lion G.N., Mdakane S.T.R. (2012). Uptake and translocation of heavy metals by medicinal plants growing around a waste dump site in Pretoria, South Africa. S. Afr. J. Bot..

[25] Tripathi S., Sharma P., Singh K., Purchase D., Chandra R. (2021). Translocation of heavy metals in medicinally important herbal plants growing on complex organometallic sludge of sugarcane molasses-based distillery waste. Environ. Technol. Innov..

[26] Pehoiu G., Murarescu O., Radulescu C., Dulama I.D., Teodorescu S., Stirbescu R.M., Bucurica I.A., Stanescu S.G. (2020). Heavy metals accumulation and translocation in native plants grown on tailing dumps and human health risk. Plant Soil.

[27] Srivastava A., Jain V.K. (2007). Size distribution and source identification of total suspended particulate matter and associated heavy metals in the urban atmosphere of Delhi. Chemosphere.

[28] Hu X., Zhang Y., Luo J., Xie M., Wang T., Lian H. (2011). Accumulation and quantitative estimates of airborne lead for a wild plant (*Aster subulatus*). Chemosphere.

[29] Hieu N.T., Lee B.-K. (2010). Characteristics of particulate matter and metals in the ambient air from a residential area in the largest industrial city in Korea. Atmos. Res..

[30] Casotti Rienda I., Nunes T., Amato F., Lucarelli F., Kováts N., Hubai K., Alves C.A. (2023). Preliminary assessment of road dust from Portuguese motorways: Chemical profile, health risks, and ecotoxicological screening. Air Qual. Atmos. Health.

[31] Khillare P.S., Balachandran S., Meena B.R. (2004). Spatial and temporal variation of heavy metals in atmospheric aerosol of Delhi. Environ. Monit. Assess..

[32] Velali E., Papachristou E., Pantazaki A., Choli-Papadopoulou T., Planou S., Kouras A., Manoli E., Besis A., Voutsa D., Samara C. (2016). Redox activity and in vitro bioactivity of the water-soluble fraction of urban particulate matter in relation to particle size and chemical composition. Environ. Pollut..

[33] Zhang Y., Ji X., Ku T., Li G., Sang N. (2016). Heavy metals bound to fine particulate matter from northern China induce season-dependent health risks: A study based on myocardial toxicity. Environ. Pollut..

[34] Tomašević M., Vukmirović Z., Rajšić S., Tasić M., Stevanović B. (2005). Characterization of trace metal particles deposited on some deciduous tree leaves in an urban area. Chemosphere.

[35] Pan Y.P., Wang Y.S. (2015). Atmospheric wet and dry deposition of trace elements at 10 sites in Northern China. Atmos. Chem. Phys..

[36] Muezzinoglu A., Cizmecioglu S.C. (2006). Deposition of heavy metals in a Mediterranean climate area. Atmos. Res..

[37] Cherednichenko V.S., Cherednichenko A.V., Cherednichenko A.V., Zheksenbaeva A.K., Madibekov A.S. (2021). Heavy metal deposition through precipitation in Kazakhstan. Heliyon.

[38] Schreck E., Foucault Y., Sarret G., Sobanska S., Cécillon L., Castrec-Rouelle M., Uzu G., Dumat C. (2012). Metal and metalloid foliar uptake by various plant species exposed to atmospheric industrial fallout: Mechanisms involved for lead. Sci. Total Environ..

[39] El-Khatib A.A., Barakat N.A., Youssef N.A., Samir N.A. (2020). Bioaccumulation of heavy metals air pollutants by urban trees. Int. J. Phytoremediat..

[40] Weerakkody U., Dover J.W., Michell P., Reiling K. (2018). Evaluating the impact of individual leaf traits on atmospheric particulate matter accumulation using natural and synthetic leaves. Urban For. Urban Green..

[41] Sæbø A., Popek R., Nawrot H., Hanslin H.M., Gawronska H., Gawronski S.W. (2012). Plant species differences in particulate matter accumulation on leaf surfaces. Sci. Total Environ..

[42] Margenat A., Matamoros V., Díez S., Cañameras N., Comas J., Bayona J.M. (2018). Occurrence and bioaccumulation of chemical contaminants in lettuce grown in peri-urban horticulture. Sci. Total Environ..

[43] Feki K., Tounsi S., Mrabet M., Mhadhbi H., Brini F. (2021). Recent advances in physiological and molecular mechanisms of heavy metal accumulation in plants. Environ. Sci. Pollut. Res..

[44] Al-Rashedy H.S.M. (2020). Effect of cobalt and nickel on growth and some physiological aspects of mint (*Mentha spicata*). Plant Cell Biotechnol. Mol. Biol..

[45] Giannakoula A., Therios I., Chatzissavvidis C. (2021). Effect of lead and copper on photosynthetic apparatus in citrus (*Citrus aurantium* L.) plants. The role of antioxidants in oxidative damage as a response to heavy metal stress. Plants.

[46] Sytar O., Kumar A., Latowski D., Kuczynska P., Strzałka K., Prasad M.N.V. (2013). Heavy metal-induced oxidative damage, defense reactions, and detoxification mechanisms in plants. Acta Physiol. Plant..

[47] Goncharuk E.A., Zagoskina N.V. (2023). Heavy metals, their phytotoxicity, and the role of phenolic antioxidants in plant stress responses with focus on cadmium. Molecules.

[48] Dinu C., Gheorghe S., Tenea A.G., Stoica C., Vasile G.G., Popescu R.L., Serban E.A., Pascu L.F. (2021). Toxic Metals (As, Cd, Ni, Pb) impact in the most common medicinal plant (*Mentha piperita*). Int. J. Environ. Res. Public Heaith.

[49] Mansoor S., Ali A., Kour N., Bornhorst J., AlHarbi K., Rinklebe J., Abd El Moneim D., Ahmad P., Chung Y.S. (2023). Heavy metal induced oxidative stress mitigation and ROS scavenging in plants. Plants.

[50] Biswas T., Parveen O., Pandey V.P., Mathur A., Dwivedi U.N. (2020). Heavy metal accumulation efficiency, growth and centelloside production in the medicinal herb *Centella asiatica* (L.) urban under different soil concentrations of cadmium and lead. Ind. Crop Prod..

[51] Hubai K., Kováts N., Sainnokhoi T.A., Eck-Varanka B., Hoffer A., Tóth Á., Teke G. (2022). Phytotoxicity of particulate matter from controlled burning of different plastic waste types. Bull. Environ. Contam. Tox..

[52] Ahmad I.Z., Ahmad A., Mabood A., Tabassum H., Khan M., Khan N. (2017). Effects of Different Metal Stresses on the Antioxidant Defense Systems of Medicinal Plants. Reactive Oxygen Species and Antioxidant Systems in Plants: Role and Regulation Under Abiotic Stress.

[53] Rahbarian R., Azizi E., Behdad A., Mirbolook A. (2019). Effects of chromium on enzymatic/nonenzymatic antioxidants and oxidant levels of *Portulaca oleracea* L.. J. Med. Plants By-Prod..

[54] Kabata-Pendias A., Pendias H. (1991). Trace Elements in Soils and Plants.

[55] Pirooz P., Amooaghaie R., Ahadi A., Sharififar F., Torkzadeh-Mahani M. (2022). Silicon and nitric oxide synergistically modulate the production of essential oil and rosmarinic acid in *Salvia officinalis* under Cu stress. Protoplasma.

[56] Es-sbihi F.Z., Hazzoumi Z., Benhima R., Amrani Joutei K. (2020). Effects of salicylic acid on growth, mineral nutrition, glandular hairs distribution and essential oil composition in *Salvia officinalis* L. grown under copper stress. Environ. Sustain..

[57] Elzaawely A.A., Xuan T.D., Tawata S. (2007). Changes in essential oil, kava pyrones and total phenolics of *Alpinia zerumbet* (Pers.) B.L. Burtt. & R.M. Sm. leaves exposed to copper sulphate. Environ. Exp. Bot..

[58] Lajayer H.A., Savaghebi G., Hadian J., Hatami M., Pezhmanmehr M. (2017). Comparison of copper and zinc effects on growth, micro-and macronutrients status and essential oil constituents in pennyroyal (*Mentha pulegium* L.). Braz. J. Bot..

[59] Babashpour-Asl M., Farajzadeh-Memari-Tabrizi E., Yousefpour-Dokhanieh A. (2022). Foliar-applied selenium nanoparticles alleviate cadmium stress. through changes in physio-biochemical status and essential oil profile of coriander (*Coriandrum sativum* L.) leaves. Environ. Sci. Pollut. Res..

[60] Fattahi B., Arzani K., Souri M.K., Barzegar M. (2019). Effects of cadmium and lead on seed germination, morphological traits, and essential oil composition of sweet basil (*Ocimum basilicum* L.). Ind. Crop Prod..

[61] Poursaeid M., Iranbakhsh A., Ebadi M., Fotokian M.H. (2021). Morpho-physiological and phytochemical responses of basil (*Ocimum basilicum* L.) to toxic heavy metal cadmium. Not. Bot. Horti Agrobot. Cluj-Napoca.

[62] Mohammed N.A., Ali W.N., Younis Z.M., Zeebaree P.J., Qasim M.J. (2024). Response of Two Mint Cultivars Peppermint (*Mentha piperita* L.) and Curly Mint (*Mentha spicata* var. *crispa*) to Different Levels of Cadmium Contamination. Pak. J. Life Soc. Sci..

[63] Youssef N.A. (2021). Changes in the morphological traits and the essential oil content of sweet basil (*Ocimum basilicum* L.) as induced by cadmium and lead treatments. Int. J. Phytoremediat..

[64] Kilic S., Kilic M. (2017). Effects of cadmium-induced stress on essential oil production, morphology and physiology of lemon balm (*Melissa officinalis* L., Lamiaceae). Appl. Ecol. Environ. Res..

[65] Amirmoradi S., Moghaddam P.R., Koocheki A., Danesh S., Fotovat A. (2012). Effect of cadmium and lead on quantitative and essential oil traits of peppermint (*Mentha piperita* L.). Not. Sci. Biol..

[66] Azimychetabi Z., Nodehi M.S., Moghadam T.K., Motesharezadeh B. (2021). Cadmium stress alters the essential oil composition and the expression of genes involved in their synthesis in peppermint (*Mentha piperita* L.). Ind. Crop Prod..

[67] Kunwar G., Pande C., Tewari G., Singh C., Kharkwal G.C. (2015). Effect of heavy metals on terpenoid composition of *Ocimum basilicum* L. and *Mentha spicata* L.. J. Essen. Oil Bear. Plants.

[68] Sulastri Y.S., Tampubolon K. (2019). Aromatic plants: Phytoremediation of cadmium heavy metal and the relationship to essential oil production. Int. J. Sci. Technol. Res..

[69] Sá R.A., Alberton O., Gazim Z.C., Laverde Jr A., Caetano J., Amorin A.C., Dragunski D.C. (2015). Phytoaccumulation and effect of lead on yield and chemical composition of *Mentha crispa* essential oil. Desalin. Water Treat..

[70] Prasad A., Singh A.K., Chand S., Chanotiya C.S., Patra D.D. (2010). Effect of chromium and lead on yield, chemical composition of essential oil, and accumulation of heavy metals of mint species. Commun. Soil Sci. Plan..

[71] Nabi A., Naeem M., Aftab T., Khan M.M.A. (2020). Alterations in photosynthetic pigments, antioxidant machinery, essential oil constituents and growth of menthol mint (*Mentha arvensis* L.) upon nickel exposure. Braz. J. Bot..

[72] Biswas S., Koul M., Bhatnagar A.K. (2015). Effect of arsenic on trichome ultrastructure, essential oil yield and quality of *Ocimum basilicum* L.. Med. Plant Res..

[73] Scora R.W., Chang A.C. (1997). Essential Oil Quality and Heavy Metal Concentrations of Peppermint Grown on a Municipal Sludge-Amended Soil. J. Environ. Qual..

[74] Pandey J., Chand S., Pandey S., Patra R.D.D. (2015). Palmarosa (*Cymbopogon martinii* (Roxb.) Wats.) as a putative crop for phytoremediation, in tannery sludge polluted soil. Ecotox. Environ. Saf..

[75] Gautam M., Agrawal M. (2017). Influence of metals on essential oil content and composition of lemongrass (*Cymbopogon citratus* (D.C.) Stapf.) grown under different levels of red mud in sewage sludge amended soil. Chemosphere.

[76] Zheljazkov V.D., Nielsen N.E. (1996). Studies on the effect of heavy metals (Cd, Pb, Cu, Mn, Zn and Fe) upon the growth, productivity and quality of lavender (*Lavandula angustifolia* Mill.) production. J. Essent. Oil Res..

[77] Gharib F.A., Mansour K.H., Ahmed E.Z., Galal T.M. (2021). Heavy metals concentration, and antioxidant activity of the essential oil of the wild mint (*Mentha longifolia* L.) in the Egyptian watercourses. Int. J. Phytoremediat..

[78] Givianrad M.H., Hashemi A.Z.A.M. (2014). A survey of the effect of some heavy metals in plant on the composition of the essential oils close to Veshnaveh-Qom mining area. Orient. J. Chem..

[79] Basile A., Botta B., Bruno M., Rigano D., Sorbo S., Conte B., Rosselli S., Senatore F. (2010). Effects of air pollution on production of essential oil in Feijoa Sellowiana Berg. grown in the ‘Italian Triangle of Death’. Int. J. Environ. Health.

[80] Judzentiene A., Stikliene A., Kupcinskiene E. (2007). Changes in the essential oil composition in the needles of Scots pine (*Pinus sylvestris* L.) under anthropogenic stress. Sci. World.

[81] Nivinskiene O., Butkiene R., Gudalevic A., Mockute D., Meskauskiene V., Grigaliunaite B. (2007). Influence of urban environment on chemical composition of *Tilia cordata* essential oil. Chemija.

[82] Hubai K., Székely O., Teke G., Kováts N. (2021). Is essential oil production influenced by air pollution in *Ocimum basilicum* L.?. Biochem. Syst. Ecol..

[83] Desalme D., Binet P., Chiapusio G. (2013). Challenges in tracing the fate and effects of atmospheric polycyclic aromatic hydrocarbon deposition in vascular plants. Environ. Sci. Technol..

[84] Figueiredo A.C., Barroso J.G., Pedro L.G., Scheffer J.J.C. (2008). Factors affecting secondary metabolite production in plants: Volatile components and essential oils. Flavour Fragr. J..

[85] Bartwal A., Mall R., Lohani P., Guru S.K., Arora S. (2013). Role of secondary metabolites and brassinosteroids in plant defense against environmental stresses. J. Plant Growth Regul..

[86] Bibbiani S., Colzi I., Taiti C., Nissim W.G., Papini A., Mancuso S., Gonnelli C. (2018). Smelling the metal: Volatile organic compound emission under Zn excess in the mint *Tetradenia riparia*. Plant Sci..

[87] Maiga A., Diallo D., Bye R. (2005). Determination of some toxic and essential metal ions in medicinal and edible plants from Mali. J. Agric. Food Chem..

[88] Chen Y.G., Huang J.H., Luo R., Ge H.Z., Wołowicz A., Wawrzkiewicz M., Gładysz-Płaska A., Li B., Yu Q.-X., Kołodyńska D. (2021). Impacts of heavy metals and medicinal crops on ecological systems, environmental pollution, cultivation, and production processes in China. Ecotoxicol. Environ. Saf..

[89] Luo L., Wang B., Jiang J., Fitzgerald M., Huang Q., Yu Z., Li H., Zhang J., Wei J., Yang C. (2021). Heavy metal contaminations in herbal medicines: Determination, comprehensive risk assessments, and solutions. Front. Pharmacol..

[90] Karahan F. (2023). Evaluation of trace element and heavy metal levels of some ethnobotanically important medicinal plants used as remedies in Southern Turkey in terms of human health risk. Biol. Trace Element Res..

[91] Asiminicesei D.M., Vasilachi I.C., Gavrilescu M.A.R.I.A. (2020). Heavy metal contamination of medicinal plants and potential implications on human health. Rev. Chim..

[92] Sarma H., Deka S., Deka H., Saikia R.R., Whitacre D. (2012). Accumulation of Heavy Metals in Selected Medicinal Plants. Reviews of Environmental Contamination and Toxicology.

[93] Collin S., Baskar A., Geevarghese D.M., Ali M.N.V.S., Bahubali P., Choudhary R., Lvov V., Tovar G.I., Senatov F., Koppala S. (2022). Bioaccumulation of lead (Pb) and its effects in plants: A review. J. Hazard. Mater. Lett..

[94] Chand S., Singh S., Singh V.K., Patra D.D. (2015). Utilization of heavy metal-rich tannery sludge for sweet basil (*Ocimum basilicum* L.) cultivation. Environ. Sci. Pollut. Res..

[95] Dinu C., Vasile G.G., Buleandra M., Popa D.E., Gheorghe S., Ungureanu E.M. (2020). Translocation and accumulation of heavy metals in *Ocimum basilicum* L. plants grown in a mining-contaminated soil. J. Soil Sediments.

[96] Lal K., Yadav R.K., Kaur R., Bundela D.S., Khan M.I., Chaudhary M., Meena R.L., Dar S.R., Singh G. (2013). Productivity, essential oil yield, and heavy metal accumulation in lemon grass (*Cymbopogon flexuosus*) under varied wastewater–Groundwater irrigation regimes. Ind. Crop Prod..

[97] Lydakis-Simantiris N., Fabian M., Skoula M. (2016). Cultivation of medicinal and aromatic plants in heavy metal-contaminated soils. Glob. Nest J..

[98] Angelova V.R., Grekov D.F., Kisyov V.K., Ivanov K.I. (2015). Potential of lavender (*Lavandula vera* L.) for phytoremediation of soils contaminated with heavy metals. Int. J. Agric. Biosyst. Eng..

